# Factors associated with acute cardiac injury and their effects on mortality in patients with COVID-19

**DOI:** 10.1038/s41598-020-77172-1

**Published:** 2020-11-24

**Authors:** Xingwei He, Luyan Wang, Hongjie Wang, Yang Xie, Yongfu Yu, Jianhua Sun, Jiangbo Yan, Yuxin Du, Yin Shen, Hesong Zeng

**Affiliations:** 1 Department of Cardiology, Tongji Hospital, Tongji Medical College, Huazhong University of Science and Technology, Wuhan, 430030 China; 2 Heart Center, Peking University Peoples Hospital, Beijing, China; 3 Department of Clinical Epidemiology, Aarhus University Hospital, Arhus, Denmark; 4 Medical Research Institute, Wuhan University Renmin Hospital, Wuhan University, Wuhan, 430060 China

**Keywords:** Biomarkers, Cardiology, Risk factors

## Abstract

To determine the incidence of acute cardiac injury (ACI), the factors associated with ACI and the in-hospital mortality in patients with COVID-19, especially in severe patients. All consecutive in-patients with laboratory-confirmed COVID-19 from Tongji Hospital in Wuhan during February 1 and March 29, 2020 were included. The demographic, clinical characteristics, laboratory, radiological and treatment data were collected. Univariate and Firth logistic regression analyses were used to identify factors associated with ACI and in-hospital mortality, and Kaplan–Meier method was used to estimate cumulative in-hospital mortality. Among 1031 patients included, 215 (20.7%) had ACI and 501 (48.6%) were severe cases. Overall, 165 patients died; all were from the severe group, and 131 (79.39%) had ACI. ACI (OR = 2.34, *P* = 0.009), male gender (OR = 2.58,* P* = 0.001), oximeter oxygen saturation (OR = 0.90, *P* < 0.001), lactate dehydrogenase (OR = 3.26, *P* < 0.001), interleukin-6 (IL-6) (OR = 8.59, *P* < 0.001), high sensitivity C-reactive protein (hs-CRP) (OR = 3.29, *P* = 0.016), N-terminal pro brain natriuretic peptide (NT-proBNP) (OR = 2.94, *P* = 0.001) were independent risk factors for the in-hospital mortality in severe patients. The mortality was significantly increased among severe patients with elevated hs-CRP, IL-6, hs-cTnI, and/or NT-proBNP. Moreover, the mortality was significantly higher in patients with elevation of both hs-cTnI and NT proBNP than in those with elevation of either of them. ACI develops in a substantial proportion of patients with COVID-19, and is associated with the disease severity and in-hospital mortality. A combination of hs-cTnI and NT-proBNP is valuable in predicting the mortality.

## Introduction

Coronavirus disease 2019 (COVID-19), caused by severe acute respiratory syndrome coronavirus 2 (SARS-Cov-2), has resulted in considerable morbidity and mortality worldwide since December 2019^1–4^.Although acute respiratory failure due to diffuse alveolar damage is the leading cause of the death from COVID-19, lungs are not the only organs involved in the disease. A few studies have found that a substantial proportion of patients with confirmed COVID-19 suffer from acute cardiac injury (ACI), which may progress to heart failure, and thus increase the risk of in-hospital mortality in some cases^2,5–8^. Furthermore, studies have shown that several inflammatory factors, such as high sensitivity C-reactive protein (hs-CRP) and interleukin-6 (IL-6), and cardiac injury indicators, such as high-sensitivity cardiac troponin I (hs-cTnI) and N-terminal pro brain natriuretic peptide (NT-proBNP), play important roles in the progression of COVID-19 and death^9^. However, the sample sizes are relatively small in these studies, and the factors associated with ACI are still not well established. Moreover, it is unclear which factors or indicators (inflammatory or cardiac) have a greater value in predicting in-hospital mortality in COVID-19 patients.

Therefore, the aims of this large retrospective study were to determine the incidence of ACI, identify factors associated with ACI and evaluate the values of potential risk factors, such as inflammatory and cardiac injury indicators, in predicting in-hospital mortality in patients with laboratory-confirmed COVID-19, especially in severe cases.

## Results

### Demographics and clinical characteristics of patients

During February 1 and March 29, 2020, a total of 1348 suspected COVID-19 patients were hospitalized at Tongji Hospital, among which 154 patients were excluded—124 patients tested negative for nucleic acid, 6 patients with incomplete clinical data and 23 patients under the age of 18. Among the remaining 1195 eligible patients, 145 had no key data (hs-cTnI) in their medical records as of March 29, 2020. 17 patients had chronic renal failure and 2 patients suffered from acute myocardial infarction during hospitalization. Thus, 1031 patients were included in the final analysis (Figure S1). Of these patients, 530 and 501 were defined as mild and severe cases, respectively. As of March 29, 2020, 165 (16.0%) patients died during hospitalization and 866 (84.0%) recovered and were discharged from hospital. The median age was 63 years (range, 24–92 years; IQR, 52–70 years), and 538 (52.2%) were male. Hypertension (37.1%) was the most common comorbidity, followed by DM (18.3%) and CHD (8.1%). The most common symptoms on admission were fever (83.3%) and dry cough (69.4%), followed by dyspnea (25.7%) and diarrhea (17.2%) (Table 1). Compared with mild patients, severe patients were older, more male-predominant, and had more comorbidities (*e.g.* hypertension, DM, CHD, and COPD) and lower oximeter oxygen saturation (OOS, all *P* < 0.05) (Table 1).

**Table 1:** Characteristics of enrolled patients and comparisons between mild and severe COVID-19 patients.

	Total (n = 1031)	Mild* (n = 530)	Severe* (n = 501)	*P* value
**Demographics and clinical characteristics**				
Age, median (IQR)-year	63 (52–70)	60 (46–67)	66 (57–73)	< 0.001
**Sex**				
Male	538 (52.2)	241 (45.5)	297 (59.3)	< 0.001
Female	493 (47.8)	289 (54.5)	204 (40.7)	< 0.001
**Comorbidity**				
Hypertension	383 (37.1)	146 (27.5)	237 (47.3)	< 0.001
Diabetes mellitus	189 (18.3)	66 (12.5)	123 (24.6)	< 0.001
CHD	83 (8.1)	28 (5.3)	55 (11.0)	0.001
COPD	39 (3.8)	6 (1.1)	33 (6.6)	< 0.001
Malignancy	29 (2.8)	11 (2.1)	18 (3.6)	0.187
Cerebrovascular disease	22 (2.1)	9 (1.7)	13 (2.6)	0.390
**Signs and symptoms**				
Fever	859 (83.3)	430 (81.1)	429 (85.6)	0.055
Dry cough	716 (69.4)	363 (68.5)	353 (70.5)	0.500
Dyspnea	265 (25.7)	71 (13.4)	194 (38.7)	< 0.001
Diarrhea	177 (17.2)	82 (15.5)	95 (19.0)	0.160
Muscle ache	137 (13.3)	74 (14.0)	63 (12.6)	0.522
Chest tightness	164 (15.9)	78 (17.8)	86 (17.2)	0.307
**Vital signs at presentation**				
Heart rate, bpm	90 (80–102)	88 (80–100)	90 (80–104)	0.396
Oximeter oxygen saturation, %	96 (92–97)	97 (96–98)	91 (85–93)	< 0.001
Systolic pressure, mmHg	130 (120–144)	129 (119–140)	133 (120–145)	0.002
**Laboratory findings**				
Leukocyte count (× 10^9^/L)	5.8 (4.5–7.7)	5.3 (4.3–6.7)	6.6 (4.9–9.2)	< 0.001
< 3.5 × 10^9^/L	89 (8.6)	53 (10.0)	36 (7.2)	< 0.001
≥ 3.5 to < 9.5 × 10^9^/L	802 (77.8)	455 (85.8)	347 (69.3)	.
≥ 9.5 × 10^9^/L	140 (13.6)	22 (4.2)	118 (23.6)	.
Lymphocyte count (× 10^9^/L)	1.1 (0.7–1.5)	1.3 (0.9–1.7)	0.8 (0.6–1.2)	< 0.001
< 1.1 × 10^9^/L	539 (52.3)	189 (35.7)	350 (69.9)	< 0.001
Platelet count (× 10^9^/L)	219.0 (164.0–288.0)	229 (180–302)	205 (149–272)	< 0.001
< 100 × 10^9^/L	46 (4.5)	2 (0.4)	44 (8.8)	< 0.001
Albumin (g/L)	35.2 (31.7–39.3)	37.6 (34.1–41.3)	32.8 (29.9–36.1)	< 0.001
< 35 g/L	352 (34.1)	13 (33.3)	339 (67.7)	< 0.001
ALT (U/L)	23.0 (15.0–39.0)	21.0 (14.0–36.0)	26.0 (17.0–40.2)	< 0.001
≥ 41 U/L	213 (20.7)	92 (17.4)	121 (24.2)	0.009
eGFR (mL/min/1.73m^2^)	91.8 (77.2–102.8)	95.2 (83.6–105.3)	87.3 (69.6–98.8)	< 0.001
≥ 90 mL/min/1.73m^2^	563 (54.6)	339 (64.2)	224 (44.7)	< 0.001
≥ 60 to < 90 mL/min/1.73m^2^	346 (33.6)	163 (30.9)	183 (36.5)	.
≥ 30 to < 60 ml/min/1.73m^2^	104 (10.1)	26 (4.9)	78 (15.6)	.
< 30 mL/min/1.73m^2^	16 (1.6)	0 (0)	16 (3.2)	.
LDH (U/L)	266.0 (204.0–369.0)	225.5 (186.3–281.8)	334.5 (248.8–481.3)	< 0.001
≥ 250 U/L	564 (54.9)	190 (36.0)	374 (74.9)	< 0.001
IL-6 (pg/mL)	6.7 (2.1–36.2)	3.4 (1.6–11.0)	21.2 (4.7–82.3)	< 0.001
≥ 7 pg/L	387 (37.5)	126 (24.0)	261 (52.1)	< 0.001
hs-CRP (mg/mL)	22.3 (2.9–72.7)	6.5 (1.2–31.1)	59.5 (16.2–111.2)	< 0.001
≥ 20 mg/L	517 (52.3)	155 (31.2)	362 (73.7)	< 0.001
D-dimer (μg/mL)	0.7 (0.4–1.7)	0.5 (0.3–1.0)	1.3 (0.7–2.6)	< 0.001
≥ 0.5 μg/mL	657 (64.0)	235 (45.3)	422 (84.2)	< 0.001
hs-cTnI (pg/mL)	5.3 (2.5–14.0)	3.3 (1.9–6.6)	9.7 (3.9–25.7)	< 0.001
ACI (≥ 99^th^percentile upper reference limit)	215 (20.9)	26 (4.9)	189 (37.7)	< 0.001
NT-proBNP (pg/mL)	124.0 (43.0–374.0)	66.0 (27.0–155.0)	281.0 (95.8–767.5)	< 0.001
≥ 486 pg/mL	257 (24.8)	40 (8.2)	215 (42.9)	< 0.001
**Imaging features**				
Ground-glass opacity or patchy shadows	1009 (97.9)	511 (96.4)	498 (99.4)	0.007
Bilateral pulmonary infiltration	959 (93.0)	468 (88.3)	491 (98.0)	< 0.001

Data are expressed as median (interquartile range) or number (%), where appropriate unless specifically indicated.

*For analysis purpose, common and mild types were defined as “mild” and severe and critically ill types of COVID-19 were defined as “severe” type in the present study.

ACI, acute cardiac injury; CHD, coronary heart disease; COPD, chronic obstructive pulmonary disease; ALT, alanine transaminase.

Severe patients had higher readings of the leukocyte count, alanine transaminase (ALT), IL-6, hs-CRP, hs-cTnI, and NT-proBNP and lower readings of the lymphocyte count and eGFR, compared with mild patients (all *P* < 0.05) (Table 1). In terms of radiologic findings, bilateral pulmonary infiltration and ground-glass opacity or patchy shadows were more commonly present in severe patients than in mild patients (all *P* < 0.01) (Table 1).

### Incidence of ACI and risk factors associated with ACI in all and severely patients

Overall, ACI, defined as hs-cTnI ≥ 99th percentile upper reference limit, occurred in 215 (20.9%) of the patients included in the study. The incidence was significantly higher in severe patients than in mild patients (37.7% *vs. *4.9%, *P* < 0.001). Compared with patients without ACI, patients who developed ACI were older, had more comorbidities (*e.g.* hypertension, DM, CHD, and COPD), lower readings of the lymphocyte count, eGFR and OOS, but higher readings of the leukocyte count, ALT, IL-6, hs-CRP, and NT-proBNP on admission (all *P* < 0.05) (Table 2). Specifically among severe patients, patients with ACI were also older (70 *vs.* 64 years, *P* < 0.001), had lower OOS (90% *vs.* 93%, *P* < 0.001), and more significant changes in inflammatory indicators (*e.g*. leukocyte count, lymphocyte count, hs-CRP, and IL-6) than those without ACI (all *P* < 0.001) (Table 2). In addition, patients with ACI had significantly lower eGFR than those without ACI (76.1 vs 91.2 mL/min/1.73 m^2^, *P* < 0.001). The proportions of patients with concomitant hypertension, CHD and COPD were higher in patients with ACI than in those without ACI (52.4% *vs*. 44.2%, 14.3% *vs*. 9.0%, and 10.1% *vs*. 4.5%, and *P* = 0.080, 0.077, and 0.024, respectively).

**Table 2:** Comparisons between patients with acute cardiac injury (ACI) and those without ACI.

Variables	All patients (n = 1031)			Severe patients (n = 501)*		
	ACI (n = 215)	Non-ACI (n = 816)	*P* value	ACI (n = 189)	Non-ACI (n = 312)	*P* value
**Demographics**						
Age, median (IQR)-yr	70 (62–77)	61 (49–68)	< 0.001	70 (62–77)	64 (54–71)	< 0.001
Male	119 (55.3)	419 (51.3)	0.282	112 (59.3)	185 (59.3)	1.000
**Major comorbidities**						
Hypertension	111 (51.6)	272 (33.3)	< 0.001	99 (52.4)	138 (44.2)	0.080
Diabetes mellitus	55 (25.6)	134 (16.4)	0.003	50 (26.5)	73(23.4)	0.455
CHD	32 (14.9)	51 (6.3)	< 0.001	27 (14.3)	28 (9.0)	0.077
COPD	20 (9.3)	19 (2.3)	< 0.001	19 (10.1)	14 (4.5)	0.024
**Vital signs at admission**						
Heart rate (bpm)	90 (80–104)	89 (80–102)	0.396	91 (80–106)	90 (80–104)	0.546
Oximeter oxygen saturation, %	90 (80–93)	96 (93–98)	< 0.001	90 (83–93)	93 (90–96)	< 0.001
Systolic pressure (mmHg)	135 (122–149)	130 (119–142)	0.002	136 (123–150)	130 (119–144)	0.017
**Laboratory findings**						
Leukocyte count (× 10^9^/L)	7.8 (5.4–11.5)	5.6 (4.4–7.0)	< 0.001	8.0 (5.9–12.4)	6.0 (4.7–8.0)	< 0.001
Lymphocyte (× 10^9^/L)	0.7 (0.5–1.0)	1.2 (0.8–1.6)	< 0.001	0.7 (0.5–0.9)	0.9 (0.6–1.3)	< 0.001
ALT (U/L)	28 (19–43)	22 (14–38)	< 0.001	28 (18–43)	25 (15–40)	0.131
eGFR (ml/min/1.73m^2^)	77.1 (56.1–91.3)	94.4 (82.3–104.4)	< 0.001	76.1 (51.6–91.4)	91.2 (80.0–103.3)	< 0.001
LDH (U/L)	420.0 (275.0–588.5)	246.0 (196.5–320.0)	< 0.001	460.0 (307.0–613.0)	303.0 (227.0–402.0)	< 0.001
hs-CRP (mg/L)	72.5 (35.5–126.2)	12.7 (2.0–55.7)	< 0.001	78 (42.9–139.5)	42.8 (8.4–96.9)	< 0.001
NT-proBNP (pg/mL)	761.0 (296.0–1548.8)	81.4 (31.0–214.0)	< 0.001	815 (302–1654)	157 (56–350)	< 0.001
IL-6 (pg/L)	59.4 (18.9–176.5)	4.8 (1.9–18.8)	< 0.001	49.6 (18.5–137.4)	7.98 (2.2–29.2)	< 0.001
**Treatments**						
Antibiotics	201 (93.5)	569 (69.7)	< 0.001	179 (94.7)	241 (77.2)	< 0.001
Antiviral treatment	176 (81.9)	726 (88.9)	0.014	151 (79.9)	292 (93.6)	< 0.001
Corticosteroids	156 (72.6)	226 (27.7)	< 0.001	146 (77.2)	135 (43.3)	< 0.001
Intravenous immunoglobin	110 (51.2)	177 (21.7)	< 0.001	102 (54.0)	88 (28.2)	< 0.001
Non-invasive mechanical ventilation	135 (62.8)	56 (6.9)	< 0.001	135 (71.4)	55 (17.6)	< 0.001
Invasive mechanical ventilation	78 (36.3)	14 (1.7)	< 0.001	78 (41.3)	14 (4.5)	< 0.001
*Outcomes*						
Mortality	131 (60.9)	34 (4.2)	< 0.001	131 (69.3)	34 (10.9)	< 0.001

Data are expressed as median (interquartile range) or number (%), where appropriate unless specifically indicated.

*For analysis purpose, severe and critically ill types of COVID-19 were defined as “severe” type in the present study.

ACI, acute cardiac injury; CHD, coronary heart disease; COPD, chronic obstructive pulmonary disease; ALT, alanine transaminase; LDH, lactate dehydrogenase; IL-6, interleukin-6.

### Risk of death of all patients and severe patients

A total of 165 (32.93%) patients, all with severe COVID-19, died at the hospital; ACI accounted for 79.39% (n = 131) of the death. The mortality rate was significantly higher in cases with ACI than in those without ACI (69.31% *vs.* 10.86%, *P* < 0.001) (Table 2). Logistic multiple regression analysis showed that ACI was an independent risk factor for the in-hospital mortality in severe patients (OR = 2.34, 95%CI 1.23–4.45; *P* = 0.009). Other risk factors included: male gender (OR = 2.58; 95%CI 1.45–4.61; *P* = 0.001), OOS (OR = 0.90, 95%CI 0.87–0.93, *P* < 0.001], LDH (OR = 3.26, 95%CI 1.89–5.63; *P* < 0.001), IL-6 (OR = 8.59, 95%CI 3.22–22.96; *P* < 0.001), hs-CRP (OR = 3.29, 95%CI 1.25–8.66; *P* = 0.016), and NT-proBNP (OR = 2.94, 95%CI 1.59–5.44; *P* = 0.001) (Table 3).

**Table 3:** Univariate and multivariate regression analyses of risk factors associated with in-hospital mortality.

Variable	Univariable OR (95% CI)	*P* value	Multivariable OR (95% CI)	*P* value
Age, year	1.06 (1.04–1.08)	< 0.001	1.02 (1.00–1.04)	0.107
Male	2.84 (1.97–4.09)	< 0.001	2.58 (1.45–4.61)	0.001
Oximeter oxygen saturation, %	0.81 (0.79–0.84)	< 0.001	0.90 (0.87–0.93)	< 0.001
**Leukocyte count, × 10**^**9**^**/L**				
≥ 9.5	9.80 (6.60–14.57)	< 0.001	1.73 (0.94–3.18)	0.079
**Lymphocyte count, × 10**^**9**^**/L**				
< 1.1	7.98 (4.97–12.80)	< 0.001	1.76 (0.90–3.45)	0.100
**LDH, U/L**				
≥ 335	13.22 (8.82–19.83)	< 0.001	3.26 (1.89–5.63)	< 0.001
**IL-6, pg/mL**				
≥ 7	25.51 (10.03–64.86)	< 0.001	8.59 (3.22–22.96)	< 0.001
**hs-CRP, mg/mL**				
≥ 20	22.48 (10.06–50.22)	< 0.001	3.29 (1.25–8.66)	0.016
**NT-proBNP, pg/mL**				
≥ 486	14.83 (10.01–21.99)	< 0.001	2.94 (1.59–5.44)	0.001
**hs-cTnI, pg/L**				
≥ 99^th^ percentile upper reference limit	13.22 (8.94–19.55)	< 0.001	2.34 (1.23–4.45)	0.009

LDH, lactate dehydrogenase; IL-6, interleukin-6.

**Figure 1:**
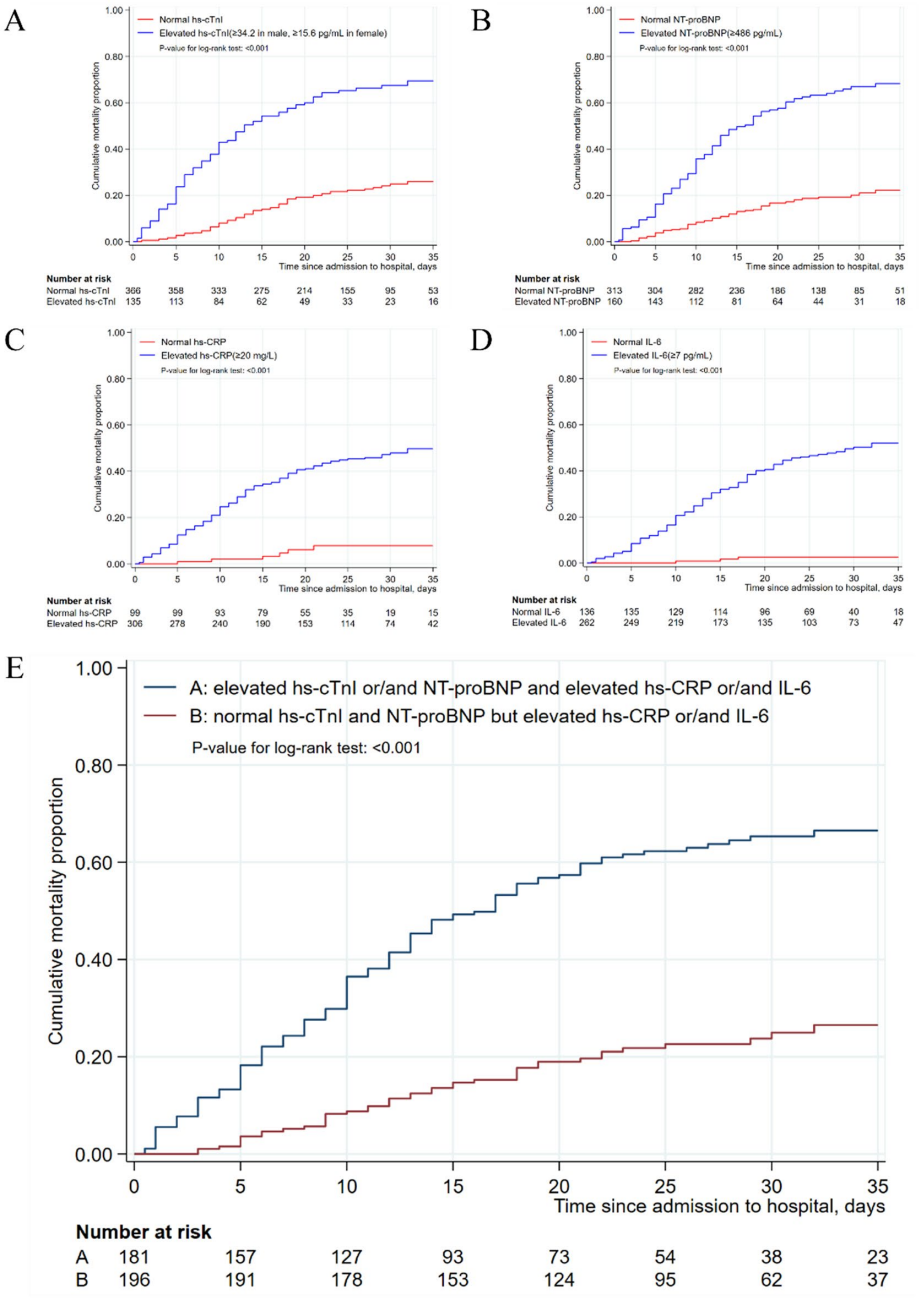
Cumulative mortality rates among severe patients with COVID-19 according to the values of hs-cTnI, hs-CRP, NT-proBNP and IL-6. (**A**) Kaplan–Meier estimates representing the probability of death in the hospital according to the value of hs-cTnI; (**B**) Kaplan–Meier estimates representing the probability of death in the hospital according to the value of NT-proBNP; (**C**) Kaplan–Meier estimates representing the probability of death in the hospital according to the value of hs-CRP; (**D**) Kaplan–Meier estimates representing the probability of death in the hospital according to the value of IL-6. (**E**) Kaplan–Meier estimates representing the comparisons of probability of death in the hospital between two groups. A: elevated hs-cTnI or/and NT-proBNP and elevated hs-CRP or/and IL-6, B: normal hs-cTnI or/and NT-proBNP but elevated hs-CRP and IL-6.

All patients, as well as severe patients, were divided into “normal” and “elevated” groups according to the values of hs-CRP, IL-6, hs-cTnI and NT-proBNP. Kaplan–Meier method was used to estimate cumulative mortality proportion between the “normal” and “elevated” groups in terms of the four individual biomarkers, and between the “normal” and “elevated” groups of cardiac injury biomarkers in severe patients with elevated inflammatory biomarkers. The log-rank test showed that, in severe patients, the cumulative mortality proportion was significantly increased in the “elevated” group, for each of the four biomarkers (all *P* < 0.001) (Fig. 1A–D). Similar results were also obtained when all patients were included in the analysis (all *P* < 0.001) (Figure S2). Moreover, the elevation in the levels of hs-cTnI or/and NT-proBNP significantly increased the in-hospital mortality in severe patients with elevated inflammatory biomarkers (*P* < 0.001) (Fig. 1E; similar results were obtained for all patients (*P* < 0.001) (Figure S2).

### Predictive value of serum hs-cTnI and NT-proBNP for cumulative in-hospital mortality among severe COVID-19 patients

**Figure 2:**
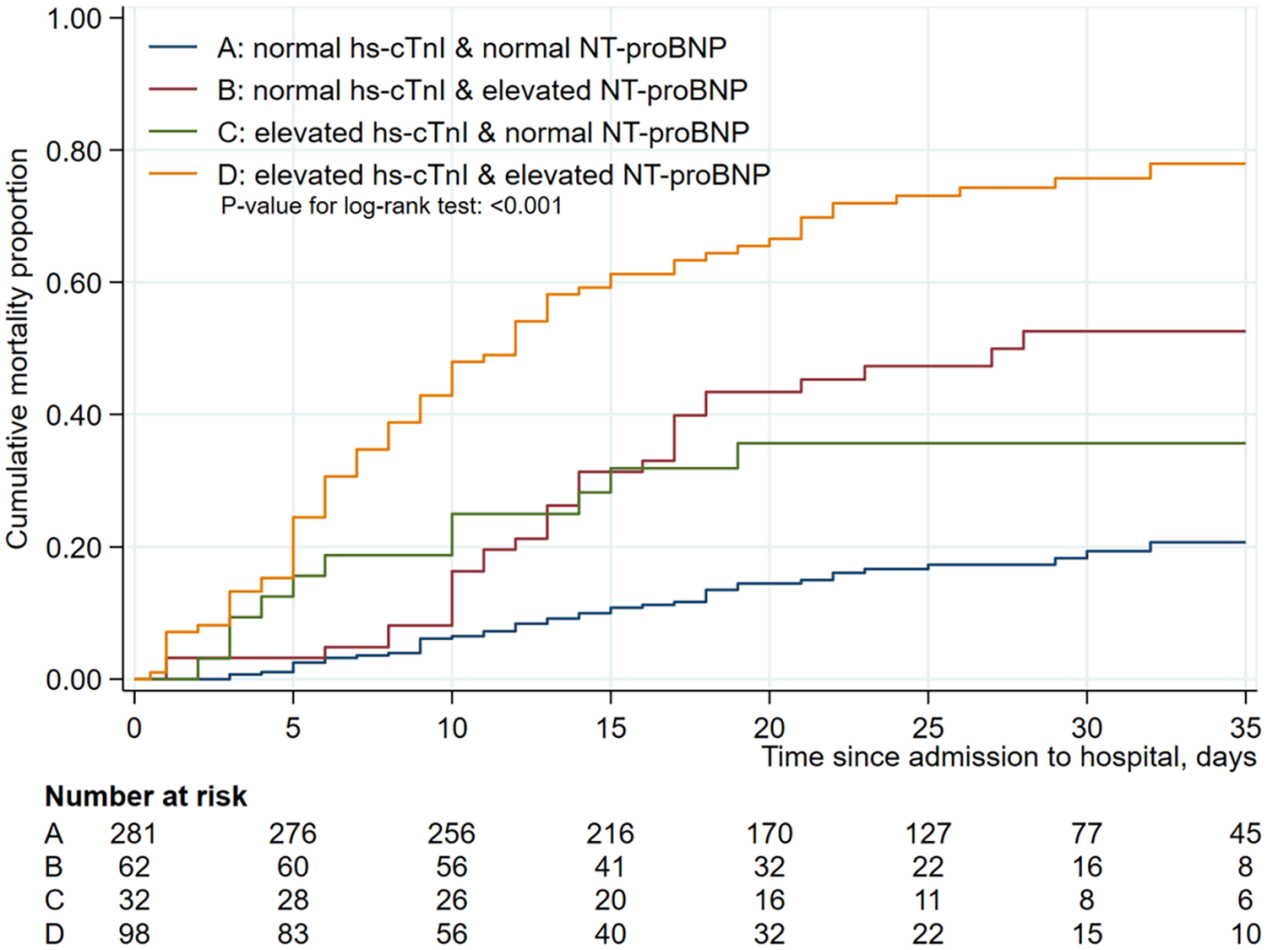
Cumulative mortality rate among hospitalized severe patients with COVID-19 according to the combination of hs-cTnI and NT-proBNP*. Kaplan–Meier estimates representing the probability of death in the hospital according to combination of hs-cTnI and NT-proBNP in severe patients. *High hs-cTnI (≥ 34.2 in male, ≥ 15.6 pg/mL in female) and high NT-proBNP (≥ 486 pg/mL).

When hs-cTnI and NT-proBNP were combined and used in Kaplan–Meier survival analysis, without consideration of the two inflammatory biomarkers, the mortality rates was the highest with elevated hs-cTnI and NT-proBNP, followed by elevated NT-proBNP and normal hs-cTnI, elevated hs-cTnI and normal NT-proBNP, and normal hs-cTnI and NT-proBNP (*P* < 0.001) (Fig. 2). Similar results were obtained for all enrolled patients (*P* < 0.001) (Figure S3).

## Discussion

In the present retrospective study, of the 1031 in-hospital patients with COVID-19 observed, 530 (51.4%) and 501 (48.6%) were mild and severe cases, respectively. ACI (defined when serum hs-cTnI was above the 99th percentile upper reference limit)^5,6^ developed in 215 (20.9%) of the patients, with the rates of 4.9% and 37.7%, respectively, in the mild and severe cases. 165 (16.0%) patients, all from severe cases, died at the hospital, and ACI was associated with accounted for 62.5% of the deaths. ACI was independently associated with in-hospital mortality of COVID-19 patients, as shown in the multivariate analysis. The elevations in the inflammatory factors, hsCRP and IL-6, and cardiac injury indicators, hs-cTnI and NT-proBNP, were associated with increased in-hospital mortality in all and severe cases, as demonstrated by the Kaplan–Meier survival analysis. Moreover, a combination of the two cardiac injury indicators, hs-cTnI and NT-proBNP, exhibited the greater value than the individual indicators alone in predicting in-hospital mortality in all and severe cases, regardless whether there was an elevation of the inflammatory factors, hsCRP and IL-6.

Our observations that ACI developed in over 20% of all cases and approximately 40% of severe cases with COVID-19, and was associated with almost 80% of the deaths suggest that ACI is a clinical condition that cannot be ignored in the course of COVID-19. Many factors may contribute to the development of ACI. In the present study, it was found that, compared with patients without ACI, patients with ACI showed notably abnormalities in many factors, including age, underlying comorbid diseases, blood oxygen saturation, inflammatory factors, kidney function, and cardiac injury indicators.

Multivariate analysis revealed that hs-CRP, IL-6, hs-cTnI, and NT-proBNP were important laboratory indicators which were independently associated with in-hospital death. HS-CRP and IL-6 are both vital inflammatory factors. While HS-CRP increases in patients with COVID-19, IL-6 promotes the secretion of antibodies by B lymphocytes and, specifically contributes to the function of natural killer lymphocytes^10,11^. Recently, IL-6 has also been shown to be associated to the immune response to the inflammatory cascade storms in the advancement of COVID-19 in various recent studies^12–14^. Thus the values of Hs-CRP and IL-6 can prove to be exceptionally pivotal in predicting in-hospital mortality. However, it has not yet been investigated thoroughly.

Hs-cTnI has been suggested as a primary indicator for determining cardiac injury, which represents the degree of myocardial injury(ACI)^15,16^, and the occurrence of ACI has been confirmed to be associated with extremely high in-hospital mortality in COVID-19 patients^6,9,17–20^, so the definition of ACI is critical to the prognosis of COVID-19 patients^21–23^. NT-proBNP represents the change of intracardial pressure, especially atrial pressure, and thus is also used as an important cardiac function indicator. In the present study, death risk analysis showed that, similar to the inflammatory indicators, abnormalities in the cardiac injury indicators, hs-cTnI and NT-proBNP, were associated with higher in-hospital mortality in all patients and in severe patients. The Kaplan–Meier curve charts demonstrated that among the patients with increased inflammatory indicators, the cumulative proportion of in-hospital mortality in patients with abnormal cardiac indicators was notably increased, which further confirmed that increased cardiac indicators had more important values than the inflammatory factors in the prognosis assessment of patients with COVID-19. Moreover, we found that the cumulative in-hospital mortality was significantly higher in patients with simultaneous increase of both hs-cTnI and NT-proBNP than inpatients with an increase of one of these indicator, suggesting that the combination of these two cardiac injury indicators would be more valuable than hs-cTnI alone in defining the presence of ACI and determining the prognosis of COVID-19 patients. Thus, based on our findings, we would propose that the definition of ACI should be not only based on hs-cTnI, but also on the cardiac function indicator, NT-proBNP. In other words, NT-proBNP should be added in the definition of ACI.

A few limitations existed in this study. First, not all laboratory studies, including IL-6 and NT-proBNP, were conducted on all patients because of the retrospective study nature. Their role in predicting in-hospital mortality may thus have been underestimated. Second, there was a shortage of data on arterial blood gas checks, electrocardiography and echocardiography in many cases due to unique circumstances during the epidemic, thereby restricting the estimation of the degree of ACI. Furthermore, this was a single-center study, and there may be selection bias. Therefore, to further validate the findings obtained in the present study, more prospective multi-centric clinical trials are required. In addition, in order to better understand the interaction between COVID-19 and ACI in the intact COVID-19 phase, thorough investigation is needed so as to optimize individual management of patients with COVID-19.

In conclusion, ACI develops in a substantial proportion of COVID-19 patients, and is associated with the disease severity and cumulative in-hospital mortality. The inflammatory factors, hs-CRP, and IL-6, and cardiac injury indicators, hs-cTnI and NT-proBNP, are all independent factors associated with the in-hospital mortality. Moreover, a combination of hs-cTnI and NT-proBNP is more valuable than the individual indicators alone in predicting the in-hospital mortality.

## Methods

### Patients and data collection

This study was conducted in accordance with the principles of the Declaration of Helsinki. The Institutional Review Board of Tongji Hospital, Wuhan, China, approved this retrospective study and written informed consent was waived (No. TJ-C20200140).

All consecutive inpatients who were hospitalized at Tongji Hospital (Wuhan China) with laboratory-confirmed COVID-19 during February 1 and March 29, 2020 were included in this retrospective study.

COVID-19 was diagnosed according to WHO interim guidance, namely, positive real-time reverse-transcriptase-polymerase-chain-reaction (RT-PCR) assay of nasal and pharyngeal swab specimens for SARS-Cov-2 nucleic acid or positive serology for anti-SARS-Cov-2 specific IgM and/or IgG antibodies.

The medical data of patients electronically recorded in the hospital system were analyzed by a research team of the COVID-19, Tongji Hospital. For each patient, demographic and clinical data, including age, sex, physical examinations, clinical manifestations, concomitant diseases, laboratory findings, radiologic information, treatment and outcomes were obtained from the electronic medical records and collected in a case report form. The data of all eligible patients were then reviewed by a trained team of physicians.

Patients were excluded if they had the following conditions: (1), unavailability of key data, such as the inflammatory and cardiac injury factors described above; (2), a history of chronic renal insufficiency (*i.e.* chronic kidney disease ≥ stage 4), and acute myocardial infarction during hospitalization; and (3) patients less than 18 year-old.

The date of disease onset was defined as the day when the symptoms of COVID-19 were noticed. Blood pressure, heart rate, and oxygen saturation were monitored regularly after admission. Initial laboratory investigations included complete blood count, liver and renal function tests, coagulation function, inflammatory biomarkers, such as hs-CRP and IL-6, and cardiac injury biomarkers, such as hs-cTnI and NT-proBNP. Chest X-ray radiograph or computed tomography (CT) scan were also performed for all inpatients, and the frequency of the examinations was determined by the treating physician. Concomitant diseases were identified as following: hypertension was diagnosed by clinical records of systolic blood pressure ≥ 140 mmHg and/or diastolic blood pressure ≥ 90 mmHg, or the use of antihypertensive agents before initial admission; diabetes mellitus (DM) was diagnosed by clinical records of fasting glucose levels ≥ 126 mg/dL, glycosylated hemoglobin A1c ≥ 6.5%, or treatment with oral hypoglycemic agents or insulin; and coronary heart disease(CHD) was defined as a previous history of myocardial infarction, or as coronary angiography showing stenosis ≥ 50% in any of the vessels, or as treatment with percutaneous coronary intervention or coronary artery bypass grafting.

### Definition of ACI and categorization of severity of COVID-19

ACI was diagnosed if the serum level of hs-cTnI was above the 99th percentile upper reference limit, regardless of new abnormalities in electrocardiography and echocardiography^2,24^. The disease severity of COVID-19 was categorized as common, mild, severe, and critically ill according to the Chinese management guideline for COVID-19 (version 6.0)^25^. For analysis purpose, common and mild types were defined as “mild” and severe and critically ill types were defined as “severe” in the present study.

### Statistical analysis

Descriptive data were expressed as mean ± standard deviation (SD) or median [range or interquartile rang (IQR)] for continuous variables, where appropriate, and frequency and percentage for categorical factors. Comparison of continuous variables was evaluated by Student’s t test or Mann–Whitney U test as appropriate. Categorical variables were compared by the Fisher exact test or χ^2^ test. Kaplan–Meier survival analysis was performed to estimate cumulative mortality rates and the log-rank test was used to determine the difference between groups. Firth logistic regression analysis was used to estimate odds ratio (OR) with 95% confidence interval (CI) to evaluate associations of demographic and clinical characteristics with COVID-19 in-hospital mortality. All analyses were conducted using SAS 9.4 (SAS Institute Inc., Cary, NC) or Stata 14 (StataCorp, College Station, TX, US).

### Ethics declarations

This study was approved by the Ethics Committee of Tongji Hospital, Wuhan, China.

## Supplementary information

Supplementary Figures.

## Data Availability

The datasets generated during and/or analyzed during the current study are available from the corresponding author on reasonable request.

## Abbreviations

ACI: Acute cardiac injury
COVID-19: Coronavirus disease 2019
hs-CRP: High sensitivity C-reactive protein
IL-6: Interleukin-6
hs-cTnI: High-sensitivity cardiac troponin I
NT-proBNP: N-terminal pro brain natriuretic peptide
DM: Diabetes mellitus
CHD: Coronary heart disease
COPD: Chronic obstructive pulmonary disease
CHD: Coronary heart disease

## References

[1] Ji Y, Ma Z, Peppelenbosch MP, Pan Q (2020). Potential association between COVID-19 mortality and health-care resource availability. Lancet Glob. Health.

[2] Huang C (2020). Clinical features of patients infected with 2019 novel coronavirus in Wuhan, China. Lancet (London, England).

[3] Livingston E, Bucher K (2020). Coronavirus disease 2019 (COVID-19) in Italy. JAMA.

[4] Cascella M, Rajnik M, Cuomo A, Dulebohn SC, Di Napoli R (2020). StatPearls.

[5] Zhou F (2020). Clinical course and risk factors for mortality of adult inpatients with COVID-19 in Wuhan, China: a retrospective cohort study. Lancet (London, England).

[6] Shi S (2020). Association of cardiac injury with mortality in hospitalized patients with COVID-19 in Wuhan, China. JAMA Cardiol..

[7] Chen C (2020). Analysis of myocardial injury in patients with COVID-19 and association between concomitant cardiovascular diseases and severity of COVID-19. Zhonghua Xin Xue Guan Bing Za Zhi.

[8] He XW, Lai JS, Cheng J, Zeng H (2020). Prognostic impact of cardiac injury in severe/critically severe patients with COVID-19. Zhonghua Xin Xue Guan Bing Za Zhi.

[9] Zhang C, Wu Z, Li J-W, Zhao H, Wang G-Q (2020). The cytokine release syndrome (CRS) of severe COVID-19 and Interleukin-6 receptor (IL-6R) antagonist Tocilizumab may be the key to reduce the mortality. Int. J. Antimicrob. Agents.

[10] Beagley KW (1989). Interleukins and IgA synthesis. Human and murine interleukin 6 induce high rate IgA secretion in IgA-committed B cells. J. Exp. Med..

[11] López-Montañés M (2017). Antibody-dependent NK cell activation differentially targets EBV-infected cells in lytic cycle and bystander B lymphocytes bound to viral antigen-containing particles. J. Immunol..

[12] Q. Ye, B. Wang, J. Mao, The pathogenesis and treatment of the `Cytokine Storm' in COVID-19. *J Infect*, (2020). 10.1016/j.jinf.2020.03.037 PMC7194613 32283152

[13] Cao X (2020). COVID-19: immunopathology and its implications for therapy. Nat. Rev. Immunol..

[14] Qin C (2020). Dysregulation of immune response in patients with COVID-19 in Wuhan, China. Clin. Infect. Dis..

[15] Shah ASV (2018). High-sensitivity troponin in the evaluation of patients with suspected acute coronary syndrome: a stepped-wedge, cluster-randomised controlled trial. Lancet (London, England).

[16] Willeit P (2017). High-sensitivity cardiac troponin concentration and risk of first-ever cardiovascular outcomes in 154,052 participants. J. Am. Coll. Cardiol..

[17] Chen T (2020). Clinical characteristics of 113 deceased patients with coronavirus disease 2019: retrospective study. BMJ.

[18] Li B (2020). Prevalence and impact of cardiovascular metabolic diseases on COVID-19 in China. Clin. Res. Cardiol..

[19] Hu Y (2020). Prevalence and severity of corona virus disease 2019 (COVID-19): a systematic review and meta-analysis. J. Clin. Virol..

[20] Guo T (2020). Cardiovascular implications of fatal outcomes of patients with coronavirus disease 2019 (COVID-19). JAMA Cardiol..

[21] Peng YD (2020). Clinical characteristics and outcomes of 112 cardiovascular disease patients infected by 2019-nCoV. Zhonghua Xin Xue Guan Bing Za Zhi.

[22] Zheng Y-Y, Ma Y-T, Zhang J-Y, Xie X (2020). COVID-19 and the cardiovascular system. Nat. Rev. Cardiol..

[23] Guzik TJ (2020). COVID-19 and the cardiovascular system: implications for risk assessment, diagnosis, and treatment options. Cardiovasc. Res..

[24] Gao C (2020). Association between cardiac injury and mortality in hospitalized patients infected with avian influenza A (H7N9) virus. Crit. Care Med..

[25] National Health Commission of the People's Republic of China. Chinese management guideline for COVID-19 (version 6.0). https://www.nhc.gov.cn/jkj/s3577/202003/4856d5b0458141fa9f376853224d41d7/files/4132bf035bc242478a6eaf157eb0d979.pdf (2020).

