# Supplementary material for: Factors associated with acute cardiac injury and their effects on mortality in patients with COVID-19
Source: Sci Rep. 2020 Nov 24;10:20452. doi: 10.1038/s41598-020-77172-1 (PMC7686361; doi:10.1038/s41598-020-77172-1)
Supplement: Supplementary file 1 — Supplementary Figures. [file 41598_2020_77172_MOESM1_ESM.docx]

**Factors associated with acute cardiac injury and their effects on mortality in patients with COVID-19**

Xingwei He ^a^, MD, Luyan Wang ^b^, MD, PHD, Hongjie Wang ^a^, PHD, MD, PHD, Yang Xie^a^ , MS , Yongfu Yu ^c^, PHD, MD, PHD, Jianhua Sun ^a^, Jiangbo Yan ^d^, MS, Yuxing Du ^d^ ,BS, Yin Shen ^d^, MD, PHD, Hesong Zeng ^a^, MD, PHD.

a. Department of Cardiology, Tongji Hospital, Tongji Medicine college, Huazhong University of Science and Technology, Wuhan, China

b. Heart Center, Peking University Peoples Hospital, Beijing, China

c. Department of Clinical Epidemiology, Aarhus University Hospital, Aarhus, Denmark

d. Medical Research Institute, Wuhan University Renmin Hospital, Wuhan University, Wuhan, China

All authors take responsibility for all aspects of the reliability and freedom from bias of the data presented and their discussed interpretation

**The first two authors contributed equally to the manuscript**

**Reprint requests and correspondence to:** Dr. Hesong Zeng, MD., PhD., Department of Cardiology, Tongji Hospital, School of Medicine, Huazhong University of Science and Technology, Wuhan 430030, China. Tel & Fax: +86-13907199959, E-mail: [zenghs@tjh.tjmu.edu.cn](mailto:zenghs@tjh.tjmu.edu.cn), Dr Yin Shen, MD., PhD., Medical Research Institute, Wuhan University Renmin Hospital, Wuhan University, Wuhan 430060, China. Tel & Fax: +86-13871550513, E-mail: [yinshen@whu.edu.cn](mailto:yinshen@whu.edu.cn).


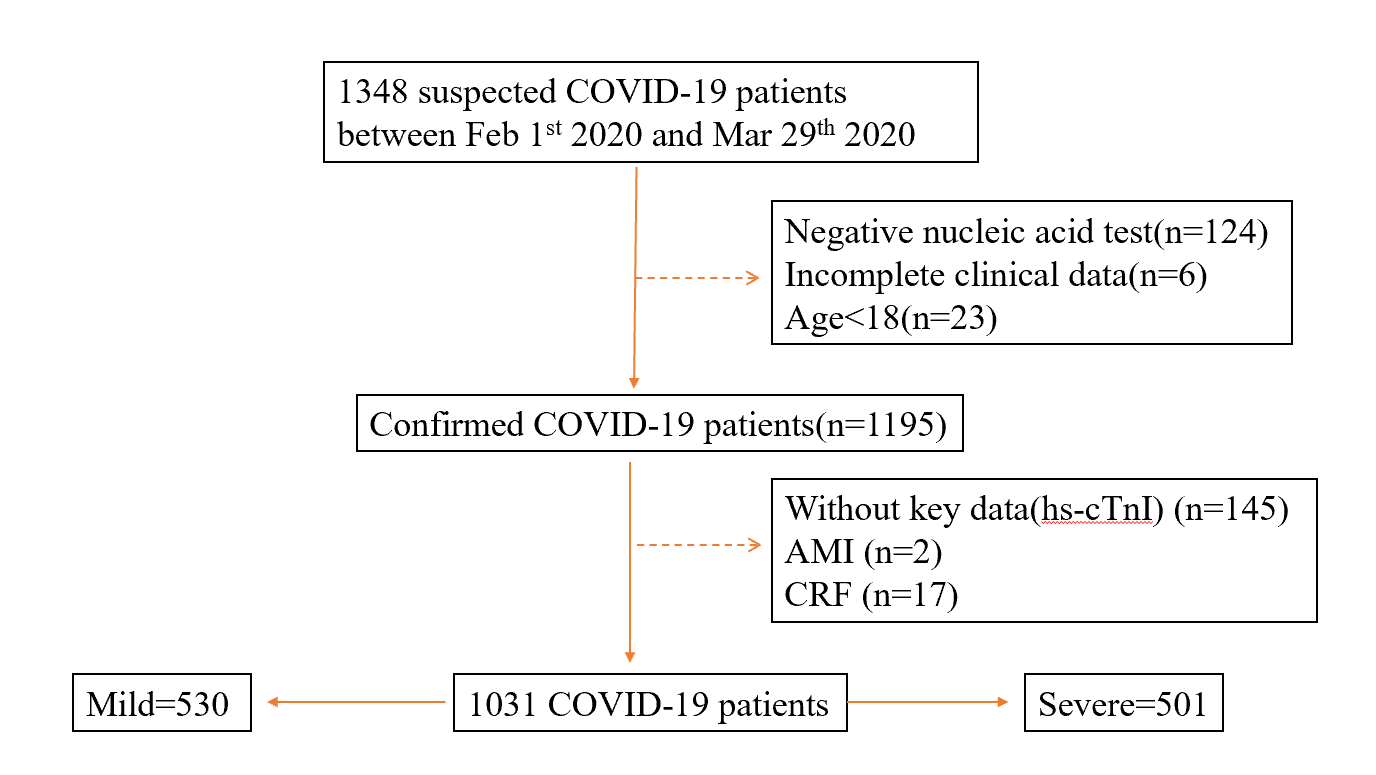


AMI: acute myocardial infarction; CRF: chronic renal failure

**Supplementary Figure S1. Study flowchart**


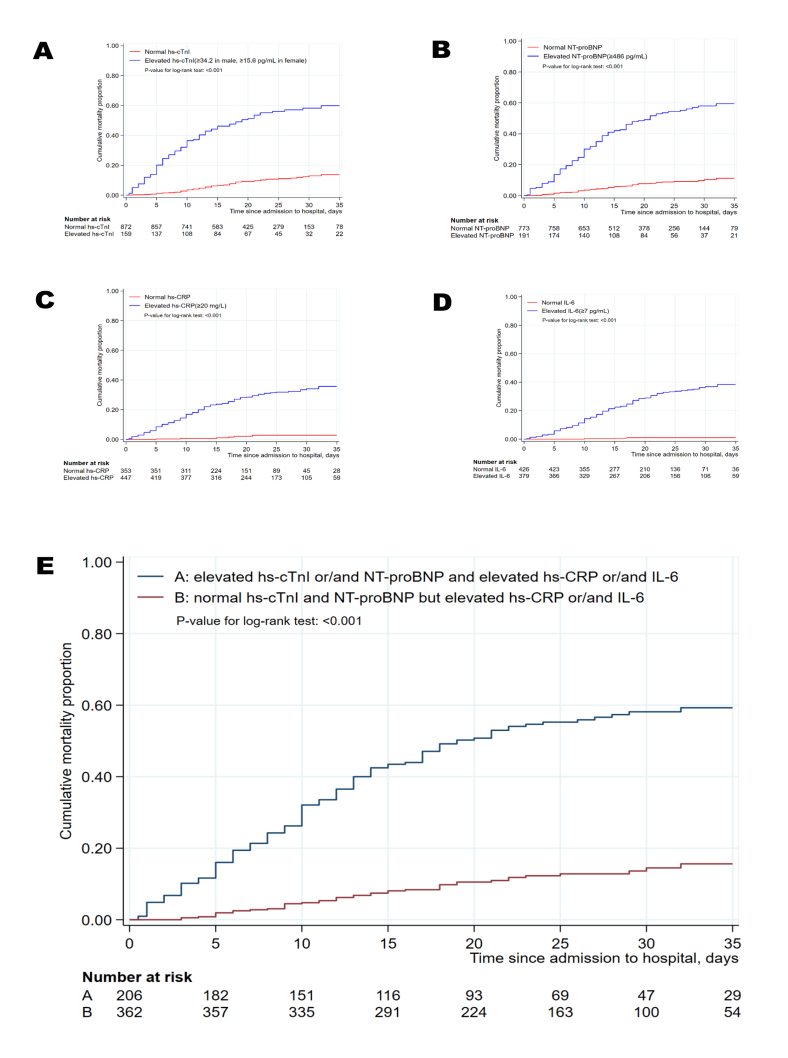


**Supplementary Figure S2. Cumulative mortality rates among all enrolled patients with COVID-19 according to the value of hs-cTnI, hs-CRP, NT-proBNP and IL-6.**

A. Kaplan-Meier estimates representing the probability of death in the hospital according to the value of hs-cTnI; B. Kaplan-Meier estimates representing the probability of death in the hospital according to the value of NT-proBNP; C. Kaplan-Meier estimates representing the probability of death in the hospital according to the value of hs-CRP; D. Kaplan-Meier estimates representing the probability of death in the hospital according to the value of IL-6; E. Kaplan-Meier estimates representing the comparisons of probability of death in the hospital between two groups. A: elevated hs-cTnI or/and NT-proBNP and elevated hs-CRP or/and IL-6, B: normal hs-cTnI and NT-proBNP but elevated hs-CRP or/and IL-6.


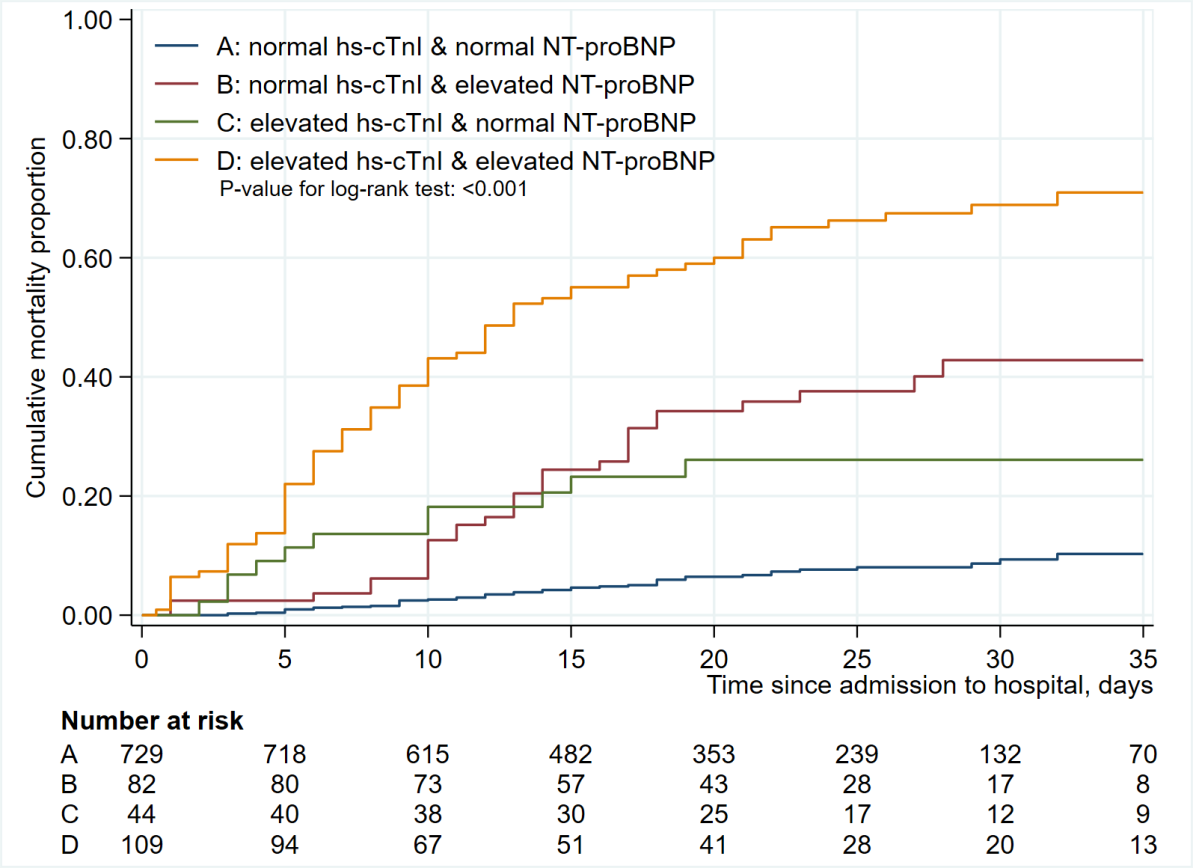


**Supplementary Figure S3. Cumulative mortality proportion among all enrolled patients with COVID-19 according to the combination of hs-cTnI and NT-proBNP*.**

Kaplan-Meier estimates representing the probability of death in the hospital according to combination of hs-cTnI and NT-proBNP in all enrolled patients.

***** High hs-cTnI (≥34.2 in male, ≥15.6 pg/mL in female), high NT-proBNP (≥486 pg/mL)
